# OA20 Pneumatosis intestinalis with spontaneous pneumoperitoneum in a patient with systemic sclerosis-myositis overlap syndrome: a case report and review of the literature

**DOI:** 10.1093/rap/rkac066.020

**Published:** 2022-09-28

**Authors:** Kian Wah Lim, Sally Knights, John Pauling, James Gotto

**Affiliations:** Department of Rheumatology, Yeovil District Hospital, Somerset, United Kingdom; Department of Rheumatology, Yeovil District Hospital, Somerset, United Kingdom; Department of Rheumatology, North Bristol NHS Trust, Bristol, United Kingdom; Department of Gastroenterology & Hepatology, Yeovil District Hospital, Somerset, United Kingdom

## Abstract

**Introduction/Background:**

Pneumatosis intestinalis (PI) is characterised by submucosal and/or subserosal collections of free gas, forming cystic lesions within the gastrointestinal (GI) tract, most commonly seen in the small intestine but it can also involve large intestine or the stomach. PI is idiopathic in about 15% of cases but the majority is secondary to conditions such as autoimmune, infectious, inflammatory, pulmonary, drug and/or traumatic aetiologies. PI is a rare and usually benign complication of systemic sclerosis (SSc), and most patients are relatively asymptomatic. Its pathogenesis is likely to involve complex interplay of mechanical, biochemical, bacterial and/or drug factors.

**Description/Method:**

A 67-year-old man with SSc-myositis overlap syndrome for over nine years on long term Mycophenolate mofetil 500mg BD and Prednisolone 8mg OD presented acutely unwell with worsening abdominal pain, bloatedness, distension, intermittent bloody diarrhoea and vomiting for three weeks. He was afebrile but slightly hypotensive and tachycardic. His abdomen was distended with mild generalised tenderness but soft and regular bowel sounds were audible. There were no palpable organomegaly, guarding, rebound tenderness or other signs of peritonism.

He had raised serum lactate at 3.1 and pre-renal acute kidney injury secondary to GI losses and reduced oral intake but his full blood count, liver blood tests, serum amylase, calcium profile, blood glucose, venous blood gas and C-reactive protein were all within normal range. His coeliac screen (IgA tTG) was normal. He tested positive for ANA Hep2 (speckled) but negative for ENA, anti-dsDNA and anti-centromere antibodies. His other SSc presentations include Raynaud’s phenomenon and diffuse scleroderma. His contrast-enhanced CT abdomen and pelvis on admission displayed extensive pneumatosis changes, moderately dilated small bowel loops and pneumoperitoneum. There are prominent atherosclerotic changes seen in aorta and its major branches with associated narrowing but there is no acute thrombotic occlusion seen in the main-stem superior or inferior mesenteric arteries or their major branches.

The patient was managed conservatively with bowel rest, intravenous fluids and antibiotics, and was referred for palliative care as surgical exploration was deemed inappropriately risky given his underlying ischaemic heart disease and impaired cardiac reserve. He was discharged home following a short hospital stay with anticipatory medications and a short course of Rifaximin followed by monthly antibiotic cycling with Co-amoxiclav, Metronidazole and Ciprofloxacin. He made a surprisingly excellent recovery on the antibiotics and his repeat CT scans at 11 weeks showed complete resolution of the pneumatosis intestinalis.

**Discussion/Results:**

PI with or without pneumoperitoneum is a rare gastrointestinal complication of SSc characterised by numerous intramural air-filled cysts within the GI tract. In spite of its usually extensive involvement of the GI tract, this condition generally runs a benign course and is managed conservatively in most cases. The development of PI in SSc is multifactorial, including mechanical (increased luminal pressure allowing gas to permeate into submucosal space through mucosal breach), biochemical (excessive hydrogen production via fermentation processes in gut), overgrowth of gas-producing bacteria and drugs such as corticosteroids that could possibly cause intestinal mucosa atrophy and breakdown.

Symptoms of PI can include diarrhoea, constipation, abdominal pain, rectal bleeding and mucous discharge. CT scan has high sensitivity and is the best diagnostic modality for PI. The treatment of PI is based on the severity of symptoms. The underlying cause of PI should be appropriately addressed and managed in all cases. Patients with asymptomatic PI need no further treatment but regular clinical review and monitoring are advisable. Conservative management is preferable to surgical intervention after careful exclusion of acute surgical causes or complications such as bowel perforation, ischaemia or obstruction. For patients with mild symptoms who do not require hospitalisation, antibiotics and elemental diet are recommended. However, for acutely unwell patients with moderate to severe symptoms, hospitalisation is usually required for bowel rest or decompression, fluid and electrolyte replacements, elemental diet or parenteral nutrition, antibiotics, high concentration oxygen, hyperbaric oxygen therapy or octreotide infusion.

Our patient was managed conservatively as he did not exhibit any clinical signs of peritonism or bowel ischaemia and surgical interventions were deemed too risky given his underlying cardiac status. He was treated with and has responded well to cycling oral antibiotics for small intestinal bacterial overgrowth which was thought to be contributory to his PI.

**Key learning points/Conclusion:**

This case report illustrates that PI could be a rare gastrointestinal complication and presentation of SSc-myositis overlap syndrome, therefore clinicians should be aware of this uncommon manifestation of SSc. Other more common differentials such as inflammatory bowel disease, infections or even an underlying malignancy must be thoroughly assessed for and excluded.

Pneumoperitoneum in the context of SSc-associated PI is generally benign in nature, spontaneous i.e. non-surgical and it occurs as a result of spontaneous rupture of the air-filled cysts within the gastrointestinal walls, rather than due to true bowel perforation. PI with or without pneumoperitoneum in patients with SSc is usually asymptomatic and only discovered incidentally on imaging or screening endoscopy. However, patients can present with more acute symptoms such as generalised abdominal pain/tenderness, distension and/or vomiting. These clinical presentations and imaging findings may simulate acute surgical abdomen such as bowel perforation and/or ischaemia, leading to diagnostic dilemma and unnecessary or inappropriately risky surgical interventions. Therefore, a precise diagnosis of PI and correct interpretation of its clinical significance are crucial, since PI is generally managed conservatively with favourable response.

Surgical treatment is generally not preferred because GI involvement in SSc is almost always extensive, and operative manipulation is likely to result in post-operative complications. However, emergency laparotomy should be considered in patients who exhibit signs of peritonism or bowel ischaemia and therefore timely involvement of surgeons and multidisciplinary team is essential.

